# Potential Roles of Prostaglandins PGD2, PGJ2, and PGE2 in the Pathogenesis of Systemic Lupus Erythematosus

**DOI:** 10.3390/ijms27114921

**Published:** 2026-05-29

**Authors:** Tian Xu, Xin M. Luo

**Affiliations:** Department of Biomedical Sciences and Pathobiology, College of Veterinary Medicine, Virginia Tech, Blacksburg, VA 24061, USA; tianxu66@vt.edu

**Keywords:** prostaglandins, systemic lupus erythematosus, inflammation, PGD2, PGJ2, PGE2

## Abstract

Prostaglandins (PGs) are eicosanoid compounds with various hormone-like effects within the human body. Prostaglandin D2 (PGD2), prostaglandin J2 (PGJ2, a metabolite of PGD2), and prostaglandin E2 (PGE2) are common prostaglandins. They are produced from arachidonic acid by cyclooxygenase isoenzymes (COX-1 and COX-2) and PG synthases and are involved in many biological metabolisms, including the inflammatory response. Recent studies show PGs are mediators for both proinflammatory and anti-inflammatory processes. In systemic lupus erythematosus (SLE), inflammation caused by autoimmunity damages tissues, leading to organ diseases such as lupus nephritis and lupus myocarditis. However, the roles of different types of PGs in the pathogenesis of SLE remain unclear. In this review, we discuss three types of PGs—PGD2, PGJ2, and PGE2—for their potential roles in SLE pathogenesis.

## 1. Introduction

Systemic lupus erythematosus (SLE) is a complex autoimmune disease characterized by dysregulated immune responses and chronic inflammation that can result in multi-organ damage [1]. Although current therapies can reduce disease activity, the mechanisms underlying immune dysregulation in SLE remain incompletely understood. Therefore, identifying pathways involved in SLE inflammation may facilitate the development of therapeutic strategies.

Prostaglandins (PGs) are lipid mediators that regulate both physiological and inflammatory processes [2,3]. Increasing evidence suggests that prostaglandins participate in immune cell regulation and inflammatory signaling pathways [4,5,6]. Among them, prostaglandin E2 (PGE2), prostaglandin D2 (PGD2), and prostaglandin J2 (PGJ2) have attracted attention because of their potential immunomodulatory roles in autoimmune and inflammatory diseases.

In this review, we first discuss the biological and immunomodulatory functions of prostaglandins, followed by the potential roles of PGE2, PGD2, and PGJ2 in SLE-associated immune dysregulation and inflammation. Because direct SLE-specific evidence remains limited in several areas, findings from related autoimmune and inflammatory diseases are also discussed to provide mechanistic insight and a framework for future investigation.

## 2. Literature Search Strategy

Relevant studies were identified through searches of the PubMed and Google Scholar databases using combinations of keywords including “systemic lupus erythematosus,” “SLE,” “prostaglandins,” “PGE2,” “PGD2,” “PGJ2,” “inflammation,” and “autoimmune disease.” Original research articles and review articles related to prostaglandin biology, immune regulation, inflammatory signaling, and autoimmune disease mechanisms published from 1964 to 2025 were considered. Studies were selected based on relevance to the scope and focus of this review.

## 3. Prostaglandins

Prostaglandins (PGs) belong to the eicosanoid family of compounds and are synthesized from arachidonic acid [7]. Prostaglandin H2 (PGH2), a precursor for specific types of PGs, is synthesized from arachidonic acid by cyclooxygenases (COX-1 and COX-2) [8]. Different types of PGs, such as prostaglandin D2 (PGD2), are converted from PGH2 by specific isomerases [8]. PGs are involved in numerous biological and metabolic functions. In muscle contraction, for example, PGs are identified as the key factors in smooth muscle contractility and regulate contraction by activating cyclic adenosine monophosphate (cAMP)-dependent K+ channels in smooth muscle cells [2]. In gastrointestinal metabolism, PGs are able to regulate gastric chloride secretion [9]. Moreover, application of PG inhibitors can influence male reproductive ability by increasing the amount of sperm, sperm motility, and sperm fertilizing capacity [10].

Other than functioning crucially in metabolic processes, PGs are also considered mediators in the occurrence of an inflammatory response. One study has shown that prostaglandin E2 (PGE2) is capable of aggravating experimental inflammatory bowel disease by mediating the expression and release of dendritic cell (DC)-derived interleukin-2 (IL-2) [3]. Another study has shown that a high level of PGD2 in epithelial mast cells can exacerbate asthma and reduce asthma control [11]. Although PGs have been well studied to function as important proinflammatory factors, there are many studies suggesting that PGs also show critical anti-inflammatory functions. In a tumor study, the role of PGD2 in tumor growth was examined, and it was suggested that PGD2 derived from the hematopoietic prostaglandin D synthase gene could suppress the inflammation caused by Lewis lung carcinoma [12]. A more recent study has demonstrated that the secretion of PGE2 by reprogrammed macrophages exhibits an anti-inflammatory effect and causes immunosuppression [13]. A similar anti-inflammatory effect on stimulated macrophages was also observed for PGE2 in a stem cell study [5]. Interestingly, prostaglandin J2 (PGJ2) primarily exhibits its anti-inflammatory properties in the presence of inflammation [6,14]. In an inflammasome study, PGJ2 was shown to inhibit caspase-1 to suppress the NLRP1 and NLRP3 inflammasomes [6]. Collectively, these studies suggest that the immunomodulatory effects of prostaglandins are highly context-dependent and may vary according to the immune microenvironment, receptor signaling, and cell types involved.

## 4. Immunopathogenesis of SLE

SLE is an autoimmune disease with dysregulated immune response and chronic inflammation that impairs organs such as the kidneys and the heart, where inflammation associated with SLE gives rise to organ-specific conditions such as lupus nephritis and lupus myocarditis, respectively [1]. These conditions resulting from SLE can ultimately lead to organ failure, which stands as the primary cause of mortality among individuals with SLE [15]. However, the exact etiology of SLE remains unclear.

An inherent feature of SLE is the presence of B cell abnormalities, particularly the heightened reactivity of B cells [16]. B cells play a significant role in the development of SLE as they actively contribute to the production of autoantibodies, which are known to worsen the progression of SLE [16]. B cells are also known to release cytokines such as IL-6 and interferon-gamma (IFNγ) [17]. These cytokines have been demonstrated to act as proinflammatory factors, increasing disease activity in individuals with SLE [18,19,20]. B cell activating factor (BAFF), a cytokine regulating B cell differentiation, is also overexpressed in patients with SLE and is believed to play a critical pathogenic role due to its ability to stimulate the autoantibody production in SLE [17,21]. Lately, a novel medication known as BAFF-Trap has emerged that has shown efficacy against SLE through the neutralization of BAFF [20].

Scientists and researchers have been actively working on finding treatments for SLE, and their efforts have resulted in the development of several treatments approved by the FDA [22]. One of them is Belimumab, which was approved in 2011 as a BAFF-targeted treatment for SLE. Another one is Saphnelo, approved by the FDA in 2021, which serves as a subsequent treatment option following Belimumab, specifically for SLE. BAFF-targeted treatments are gaining increasing popularity in the realm of SLE drug development. These treatments specifically focus on inhibiting BAFF and are becoming more widely recognized as potential therapeutic options for SLE.

In addition to BAFF, eicosanoid compounds such as PGE2 and PGD2 also function importantly in the pathogenesis of SLE, especially in the inflammatory process. Understanding the involvement of different PGs in the mechanisms of SLE will not only provide a deeper understanding of the disease pathogenesis but also promote the development of novel and effective treatments for SLE. The following sections review current evidence regarding the potential roles of different prostaglandins in SLE-associated immune dysregulation and inflammation.

## 5. Role of PGE2 in SLE

Cytosolic PGE2 synthase (cPGES) and prostaglandin E synthase (PGES) convert PGH2 into PGE2 [8,23]. PGE2 is one of the amplest PGs existing in the human body and is believed to exert both proinflammatory and anti-inflammatory roles. In this section, we will primarily discuss its proinflammatory role in SLE by focusing on T cells, B cells, and cytokines.

T cells are considered to play an important function in the pathogenesis of SLE. The secretion of proinflammatory cytokine IL-17 by T helper 17 (Th17) cells promotes inflammation and elevates the disease [24,25]. Regulatory T (Treg) cells, on the other hand, preserve the immunological self-tolerance, whereas the imbalance of Treg cells could lead to uncontrolled immune responses [26,27]. In SLE, the dysregulation of Th17/Treg balance exacerbates organ inflammation and elevates disease activity [24,28,29,30]. PGE2 directly enhances the development and proinflammatory functions of human and murine Th17 cells [31]. Mechanistically, PGE2 promotes the expression of Th17 cytokines through PGE2 receptor 2 (EP2) signaling and suppresses anti-inflammatory cytokine IL-10 produced by PGE2 receptor 4 (EP4) signaling [31]. In a multiple sclerosis study, it was shown that PGE2 could induce pathogenic Th17 cells by increasing EP2 signaling, where Th17 cells from patients induced by EP2 signaling showed higher expression of IFNγ [32]. Although multiple sclerosis and SLE are distinct autoimmune diseases, both involve dysregulated T cell responses and abnormal Th17-associated inflammation [33,34]. Therefore, these findings in multiple sclerosis suggest that PGE2-mediated EP2 signaling may also contribute to inflammatory pathways relevant to SLE pathogenesis. Another study reported that PGE2 in combination with IL-23 promoted the expansion of Th17 cells [35]. Consistently, a third study suggested that PGE2 amplified IL-23-mediated expansion of Th17 cells through EP4 signaling in T cells and dendritic cells [36]. While these studies were not performed in the context of SLE, it is reasonable to speculate that PGE2 may induce similar Th17 pathways in SLE to exacerbate disease activity.

Besides Th17 cells, Treg cells play a crucial role in maintaining self-tolerance and precluding autoimmune diseases [37,38,39]. In a study on allergic rhinitis, PGE2 was shown to suppress the differentiation of Treg cells by impacting EP4 signaling [40]. In addition, signaling of PGE2 through EP2 and EP4 receptors has been shown to disrupt the signaling of transforming growth factor-beta (TGFβ) during the differentiation of inducible Treg cells [41]. Moreover, a gut microbiota study has revealed that PGE2 facilitates intestinal inflammation by suppressing microbiota-dependent Treg cells, where the PGE2-EP4-microbiota-Treg pathway was recommended as a potential therapeutic target against intestinal inflammation [42].

**Figure 1:**
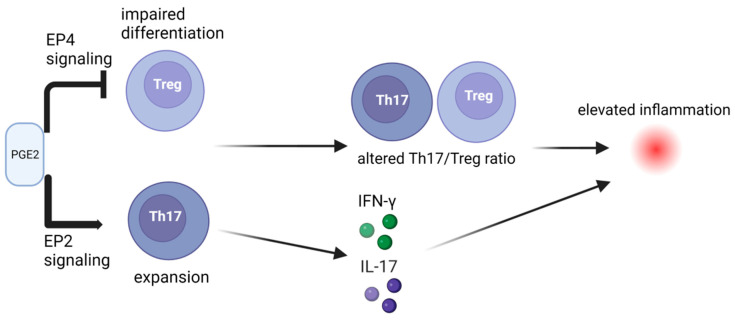
The hypothesized mechanism of PGE2 in promoting T cell-mediated inflammation in SLE. The development of Th17 cells is elevated by PGE2-stimulated EP2 signaling, and the secretion of proinflammatory cytokines, IFNγ and IL-17, is elevated. The differentiation of Treg cells is impaired by PGE2-stimulated EP4 signaling, resulting in an increased Th17/Treg ratio. The increased Th17/Treg ratio, combined with more proinflammatory cytokines, leads to elevated inflammation that may exacerbate SLE disease progression.

Based on the proinflammatory function of PGE2 to disrupt the Th17/Treg balance, we speculate that PGE2 may function to promote SLE disease development, as shown in Figure 1.

**Figure 2:**
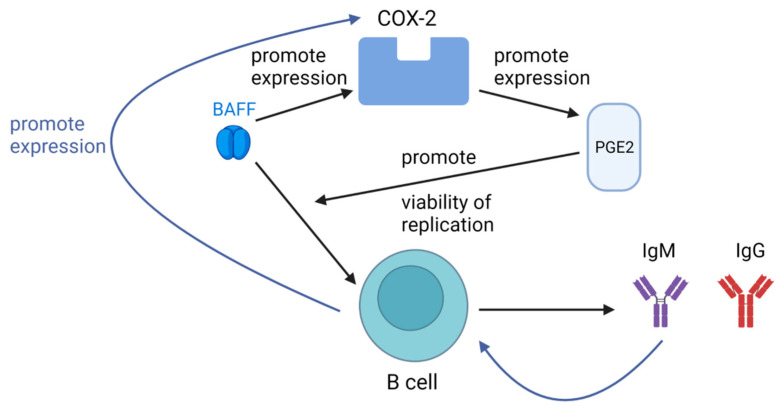
The hypothesized positive-feedback mechanism of PGE2 on B cells in the pathogenesis of SLE. Black arrows represent BAFF promoting COX-2 expression to generate PGE2 to facilitate the viability of B cell replication that leads to the generation of autoantibodies, including IgM and IgG. Blue arrows represent IgM stimulating B cells to promote COX-2 expression to generate PGE2 to participate in the mechanism.

While the dysregulation of T cells is one of the crucial components in the pathogenesis of SLE, a potentially more important abnormality within SLE is the dysregulation of B cells [16,43,44]. In SLE disease progression, B cells play a pivotal role by secreting autoantibodies and proinflammatory cytokines and generating immune complexes [45,46]. Thus, it is interesting to discuss how PGE2 might affect B cells to modulate SLE pathogenesis. Firstly, the application of COX-2 inhibitors to human B lymphocytes reduces their terminal differentiation into antibody-secreting plasma cells [47]. More specifically, COX-2 inhibition diminishes antibody isotype switching to immunoglobulin G (IgG) and dampens the generation of CD38+ IgG+ antibody-secreting cells [47]. Secondly, BAFF has been shown to increase the viability of replicating human B2 cells through promoting COX-2 expression and the production of PGE2 [48]. In the same study, it was also demonstrated that exogenous PGE2 could promote the viability and recovery of lymphoblasts [48]. These studies, together with an earlier study where B cells were shown to upregulate COX-2 and PGE2 expression in response to IgM ligation and proinflammatory signals from IFNγ [49], suggest that PGE2 has the potential to assist BAFF in increasing the viability and differentiation of B cells into antibody-secreting cells, which may contribute to the inflammation in SLE, as shown in Figure 2. While IgG directly contributes to the induction of tissue inflammation [50,51], overexpression of IgM could potentially exacerbate inflammation through promoting COX-2 to generate more PGE2. However, the exact role of IgM in the pathogenesis of SLE remains unclear, as some studies have reported that it may play a protective role [52,53].

Next, we will discuss how PGE2 might affect cytokines to modulate SLE pathogenesis. IL-6 is a cytokine that can function to promote inflammation [54]. In SLE, IL-6 can serve as a biomarker to detect disease activity in patients due to its high level in the patient’s serum [55]. Blockade of IL-6 results in attenuation of SLE disease activity [56]. IL-10, on the other hand, is an anti-inflammatory cytokine [19]. It has been shown that IL-10 can inhibit pathogenic T helper 1 (Th1) cytokine responses to attenuate murine lupus [57]. However, the actual role of IL-10 is controversial, as some studies reported high levels of IL-10 in patients with SLE, and that IL-10 can facilitate direct differentiation of activated B cells into plasma cells [58,59]. IFNγ, on the other hand, is a cytokine produced by various types of immune cells; it elevates the inflammatory process in SLE and shows abnormally high levels in the patient’s serum [60,61,62].

These three cytokines, IL-6, IL-10, and IFNγ, could be impacted by PGE2 during the progression of SLE. One study has suggested that endogenous PGE2 dysregulates the production of IL-6, IL-10, and IFNγ in pristane-induced lupus, where the splenic levels of these cytokines were extremely high compared to the control [63]. The application of indomethacin, an inhibitor of PGE2 synthesis, significantly decreased macrophage production of IL-6 and IL-10 [63]. PGE2 has been demonstrated to directly enhance IL-6 production in macrophages and monocyte-derived cells via cAMP-dependent signaling [64,65]. PGE2 also promotes IL-6 expression in human fibroblasts through EP2/EP4 receptor signaling [66]. While the literature is limited, the known function of IL-6, IL-10, and IFNγ may indicate their potential roles to mediate the effects of PGE2 on SLE pathogenesis.

Collectively, current evidence suggests that PGE2 may exert context-dependent immunomodulatory effects in SLE. However, several proposed mechanisms are derived from studies on non-SLE inflammatory or autoimmune diseases, and findings across experimental systems remain inconsistent. In addition, differences between murine models and human SLE may limit direct translational interpretation. Therefore, further SLE-specific mechanistic studies are necessary to clarify the precise role of PGE2 signaling in SLE pathogenesis.

## 6. Role of PGD2 in SLE

PGD2 is known for maintaining homeostatic functions and is converted from PGH2 by the PGD2 D-isomerase (PTGDS) [8]. PGD2 is produced by mast cells and can stimulate the generation of proinflammatory T helper 2 (Th2) cytokines, leading to activation of eosinophils [67,68]. Therefore, PGD2 has been extensively studied in the context of allergic diseases such as asthma and allergic inflammation [4,69]. In this section, we discuss how PGD2 might influence inflammation in SLE.

Compared to the proposed role of PGE2, the inflammatory role of PGD2 in SLE has some evidential support. A recent study has shown that PGD2 can accelerate lupus disease by accumulating basophils in lymphoid organs and inducing the externalization of C-X-C chemokine receptor type 4 (CXCR4) [70]. Basophils are involved in the lupus nephritis disease development, and the activation of basophils promotes SLE pathogenesis [71,72]. CXCR4, on the other hand, is overexpressed on SLE B cells, and its expression is positively correlated with SLE disease activity [73]. Moreover, in patients with SLE, Pellefigues et al. observed that both the concentration of PGD2 and the expression of PGD2 receptor (PTGDR) increased in the patient’s plasma [70]. Mechanistically, it was suggested that PGD2 upregulation in lymphoid organs contributed to basophil accumulation, leading to the exacerbation of SLE disease. Furthermore, organ damage caused by SLE could be reduced by targeting the PTGDR with specific inhibitors. Together, these results indicate that PGD2 can promote the development of SLE disease and that the application of PTGDR antagonists would lead to the improvement of organ damage, suggesting that targeting PGD2 signaling might be a novel treatment strategy against SLE [70].

Interestingly, PGD2 is also able to exert anti-inflammatory effects [12]. Mast cell–derived PGD2 limits tumor necrosis factor-alpha (TNF-α) production, vascular permeability, and inflammatory cytokine expression, thereby restraining inflammation and regulating the local immune microenvironment [12]. Although PGD2 signaling has been associated with inflammatory responses and tissue injury in SLE models, the available evidence remains relatively limited, particularly in human SLE studies. Furthermore, PGD2 may exhibit both pathogenic and protective functions depending on the immune microenvironment and receptor context. Further investigations into the complex roles of PGD2 in SLE should offer more insights prior to the establishment of PGD2 antagonists as a new therapy to treat organ damage in SLE.

## 7. Role of PGJ2 in SLE

The production of PGJ2, also known as 15d-PGJ2, relies on the enzymatic mechanisms responsible for the generation of PGD2, as 15d-PGJ2 is a derivative of PGD2 [14]. The most recently discovered PG, PGJ2, has not been well studied, although several reports have shown its potential anti-inflammatory role [14,74]. In this section, we briefly discuss the anti-inflammatory property of PGJ2 in SLE.

One study on lupus nephritis showed that PGJ2 blocked the generation of nitric oxide (NO), a proinflammatory mediator, in mesangial cells from MRL/*lpr* mice [75]. In addition, cells from MRL/*lpr* mice exhibited impaired production of PGJ2, which was associated with heightened activation of mesangial cells that promoted inflammation [75]. A second study by the same research group investigated the mechanism of how PGJ2 inhibited NO production and revealed downregulation of the expression of inducible nitric oxide synthase (iNOS) in mesangial cells through a peroxisome proliferator-activated receptor gamma (PPAR-γ)-mediated mechanism [76]. These two studies suggest that PGJ2 exerts an anti-inflammatory effect in the pathogenesis of SLE-associated kidney inflammation; however, further investigations are necessary to reveal its role in systemic lupus.

PGJ2 exerts its biological activity mainly through binding to PPAR-γ [14]. T cells are known to express PPAR-γ, and activation of PPAR-γ modulates T cell activation and differentiation [77]. One study has shown that PPAR-γ selectively inhibits the differentiation of Th17 cells in a T cell-intrinsic manner, reducing IL-17 expression [78]. In an experimental allergic encephalomyelitis study, activation of PPAR-γ by agonists impairs IFN-γ production and Th1 polarization in T cells [79]. Moreover, activation of PPAR-γ is known to enhance Treg cell responses, in part by influencing metabolic and signaling pathways that support Forkhead Box P3 (FOXP3) expression [80]. These data strongly suggest a potential anti-inflammatory effect of PGJ2 in SLE by inhibiting Th1 and Th17 while promoting Treg differentiation.

In B cells, PPAR-γ functions as an inhibitory regulator of activation and survival. Activation of PPAR-γ with PGJ2 suppresses B cell proliferation and induces apoptosis [81]. Genetic evidence further supports a restraining role for this pathway, as PPAR-γ haploinsufficiency leads to exaggerated B cell proliferative responses and enhanced antigen-specific antibody production in vivo [82]. These data suggest that PGJ2 may protect against SLE by inhibiting the differentiation and function of autoreactive B cells.

In DCs, engagement of PPAR-γ modulates maturation and function, generally shifting them toward a more tolerogenic phenotype [83,84]. Activation of PPAR-γ inhibits the upregulation of costimulatory molecules, including CD80/CD86 during DC maturation and suppresses the production of proinflammatory cytokines such as IL-12 [83,84]. Macrophages also express PPAR-γ, and its activation promotes an anti-inflammatory phenotype [85]. Activation of PPAR-γ by PGJ2 inhibits expression of iNOS, gelatinase B, and scavenger receptor A [85,86]. PPAR-γ inhibits inflammatory gene expression in part through antagonism of the transcription factors activator protein-1 (AP-1), signal transducer and activator of transcription (STAT), and nuclear factor kappa-light-chain-enhancer of activated B cells (NF-κB), indicating that PGJ2 may function as an endogenous PPAR-γ ligand in macrophages and participate in the regulation of inflammatory responses [85]. In addition, PGJ2 has been shown to inhibit activation of the NLRP3 inflammasome, thereby limiting IL-1β maturation [6].

In addition to its effects on adaptive immune regulation, PGJ2 has also been shown to directly suppress inflammatory signaling pathways through PPAR-γ-dependent and PPAR-γ-independent mechanisms. One study demonstrated that PGJ2 inhibits NF-κB activation through covalent modification of signaling proteins involved in inflammatory transcriptional regulation [87]. This finding provides additional experimental evidence supporting the potential anti-inflammatory role of PGJ2 signaling in SLE.

**Figure 3:**
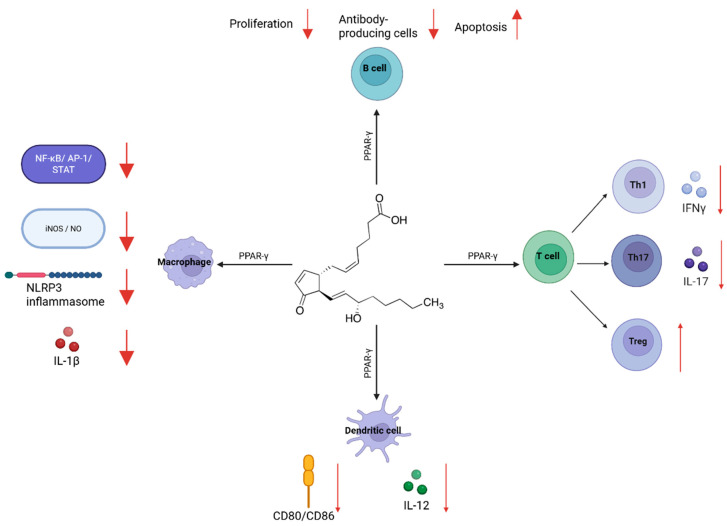
Hypothesized immunoregulatory roles of PGJ2–PPAR-γ signaling in SLE. Black arrows indicate activation of PPAR-γ by PGJ2 in immune cells. PPAR-γ signaling suppresses Th1 and Th17 responses while promoting Treg cells, limits B cell proliferation and the generation of antibody-producing cells, inhibits DC costimulatory molecule expression and IL-12 production, and attenuates macrophage inflammatory signaling and NLRP3 inflammasome activation, collectively restraining lupus-associated immune activation.

We hypothesize that PGJ2 functions as an endogenous immunoregulatory mediator that restrains lupus-associated immune dysregulation (Figure 3). Specifically, through activation of PPAR-γ, PGJ2 is predicted to suppress pathogenic Th1 and Th17 responses while enhancing Treg cell activity, thereby helping to correct the Th17/Treg imbalance characteristic of SLE. In B cells, activation of PPAR-γ by PGJ2 may directly limit B cell proliferation and survival. In DCs, PGJ2-dependent activation of PPAR-γ may contribute to a tolerogenic phenotype marked by reduced costimulatory molecule expression and diminished IL-12 production, which may attenuate pathogenic T-cell priming. In macrophages, PGJ2–PPAR-γ signaling suppresses inflammatory gene expression by antagonizing AP-1, STAT, and NF-κB activity, inhibits iNOS and matrix-degrading enzymes, and potentially limits NLRP3 inflammasome activation, thereby reducing IL-1β maturation. Collectively, these coordinated effects suggest that PGJ2–PPAR-γ signaling may act as a broad anti-inflammatory axis capable of dampening both adaptive and innate immune activation in SLE.

Although current evidence suggests that PGJ2 may exert anti-inflammatory effects through PPAR-γ signaling, the available literature in the context of SLE remains relatively limited. In addition, many proposed mechanisms are derived from experimental systems outside of SLE models. Therefore, further studies are necessary to clarify the precise role of PGJ2 in SLE-associated immune dysregulation and to determine its potential translational relevance.

## 8. Concluding Remarks

Although the exact roles of PGE2, PGD2, and PGJ2 in SLE are not yet well defined, several studies support predominantly proinflammatory roles for PGE2 and PGD2, while PGJ2 being anti-inflammatory, in the pathogenesis of SLE. However, some studies indicated just the opposite, at least for PGD2, that its role in SLE might be anti-inflammatory instead. This may be because the disease progression is long for SLE, and the roles of PGD2 may be disease stage-dependent. Further investigations are required to interpret the pro- and/or anti-inflammatory roles of PGE2, PGD2, and PGJ2 in the pathogenesis of SLE. For example, it might be meaningful to study how PGE2 is involved in the pathogenesis of lupus nephritis, one of the major organs damaged in SLE, since high levels of PGE2 have been reported in patients with lupus nephritis [88].

Another interesting point to investigate is the involvement of PGs in SLE-associated gut microbiota. Interestingly, PGE2 can facilitate the disruption of the communication between gut microbiota and Treg cells, thereby promoting inflammation in the intestines [42]. Future studies can be focused on how PGs change the gut microbiota to impact Treg cells, and whether the proinflammatory functions of PGE2 and PGD2, as well as the anti-inflammatory effect of PGJ2, in SLE are mediated by the gut microbiota.

Pharmacological modulation of prostaglandin-associated inflammatory pathways has also been explored clinically in autoimmune and inflammatory diseases. For example, selective cyclooxygenase-2 (COX-2) inhibitors such as celecoxib have been evaluated in clinical studies involving rheumatoid arthritis and juvenile idiopathic arthritis, reflecting the translational potential of targeting prostaglandin-related signaling pathways [89,90]. In addition, modulation of eicosanoid-associated inflammatory pathways through omega-3 fatty acid supplementation has also been investigated in patients with SLE [91]. However, direct prostaglandin-targeted therapies in SLE remain limited. Although several autoimmune and inflammatory diseases share dysregulated inflammatory and immune signaling pathways, important differences in disease pathogenesis, immune cell involvement, and organ-specific manifestations may limit the direct translation of findings from other autoimmune conditions to SLE. In addition, the context-dependent immunological effects of prostaglandin signaling continue to present challenges for clinical application.

Interpreting the roles of PGE2, PGD2, and PGJ2 in SLE may also facilitate the development of novel treatments against SLE. As mentioned earlier, antagonists against the PGD2 receptor have been shown to improve organ damage in patients with SLE [70]. PGE2 might be a good therapeutic target as well, whereas PGJ2, due to its anti-inflammatory role, may become a potential SLE treatment. Importantly, several mechanisms discussed in this review are supported primarily by studies performed in related autoimmune or inflammatory diseases rather than directly in SLE models. However, many hypotheses raised by this review would need to be validated before we can make clinical recommendations to benefit patients.

## Data Availability

No new data were generated or analyzed in this study. Data sharing is not applicable to this article.
